# The clinical relevance of broad mutational screening of myeloproliferative neoplasms at diagnosis

**DOI:** 10.3389/fonc.2023.1190305

**Published:** 2023-08-11

**Authors:** Helna Pettersson, Jenni Adamsson, Peter Johansson, Staffan Nilsson, Lars Palmqvist, Björn Andréasson, Julia Asp

**Affiliations:** ^1^ Hematology Section, Department of Medicine, NU Hospital Group, Uddevalla, Sweden; ^2^ Department of Laboratory Medicine, University of Gothenburg, Gothenburg, Sweden; ^3^ Hematology and Coagulation Section, Department of Medicine, Sahlgrenska University Hospital, Gothenburg, Sweden; ^4^ Department of Clinical Chemistry, Sahlgrenska University Hospital, Gothenburg, Sweden

**Keywords:** MPN, *JAK2*, *CALR*, *MPL*, germline, mutation, prognosis

## Abstract

**Introduction:**

Myeloproliferative neoplasm (MPN) is a heterogenous group of hematological malignancies including polycythemia vera (PV), essential thrombocythemia (ET) and primary myelofibrosis (PMF). *JAK2*V617F is the most frequent driver mutation in all three entities, but in PMF and ET mutations in *CALR* and *MPL* are also frequent. Mutations seen in additional genes are also often the same regardless of subtype of MPN. The aim of this study was to analyze a population based MPN cohort for genetic variants with prognostic value that can guide clinical decisions.

**Methods:**

MPN patients from Western Sweden diagnosed between 2008-2013 (n=248) were screened for mutations in 54 genes associated with myeloid malignancy.

**Results:**

Mutations in the genes *SRSF2* and *U2AF1* correlated significantly with impaired overall survival but did not correlate to increased risk for vascular events, neither before nor after diagnosis. Rather, mutations in these genes showed an association with disease transformation. Several recurrent gene variants with allele frequency close to 50% were confirmed to be germline. However, none of these variants was found to have an earlier onset of MPN.

**Discussion:**

In conclusion, we identified gene mutations to be independent markers of impaired survival in MPN. This indicates the need for more individualized assessment and treatment of MPN patients and a wider gene mutation screening already at diagnosis. This could ensure the identification of patients with high-risk mutations early on. In addition, several genetic variants were also identified as germline in this study but gave no obvious clinical relevance. To avoid conclusions from non-informative genetic variants, a simultaneous analysis of normal cell DNA from patients at diagnosis should be considered.

## Introduction

Polycythemia Vera (PV), Essential Thrombocythemia (ET) and Primary Myelofibrosis (PMF) all belong to the Philadelphia chromosome negative myeloproliferative neoplasm (MPN) category. These three entities share the same characteristics of causing proliferation of bone marrow cells, resulting in an increase of blood cells of myeloid lineage in the bone marrow and in peripheral blood. Advanced stages of PMF, on the other hand, is characterized by increase of reticulin fibers leading to decreased blood cells (1–3). The complications of these three entities are also similar regarding vascular events, i.e., thrombosis and bleeding. Furthermore, all three entities can transform into acute leukemia and have an impact on survival, however with large differences in frequency. Despite the common driver mutations in *JAK2, CALR* and *MPL* (4), the clinical presentation, risk and frequencies of complications and survival differ wildly between individual patients. Prognostic tools are therefore desired in clinical practice for follow-up and treatment decisions already at diagnosis. Modern sequencing techniques have given the opportunity to simultaneously analyze several mutations in blood malignancies. It has become widely used in both research and clinical practice (5). We and other groups have published data on risk mutations using this approach, but several studies analyze data on separate MPN entities or driver mutation groups. Since both driver mutations and several of the additional mutations found are shared between the subtypes of MPN, and since the occurrence of some additional mutations are rather rare, we hypothesize that analysis of mutations in MPN as one group has a potential to extend the prognostic value of genetic markers. Furthermore, there is a growing number of hereditary gene variants that have been linked to predisposition for development of hematological disorders, including MPN (6–11). This also represents a challenge when analyzing large amount of sequencing data, especially if comparison with normal non-malignant cells is not available. We also need to get more information regarding gene variants of unknown significance, to avoid overestimation of their importance but also to identify variants that might influence disease development and prognosis. In this study, we analyzed a well-defined, population based MPN cohort, regardless of subtype, for genetic variants. The aim was to search for additional prognostic markers that can be used to guide clinical decisions, as well as to investigate the potential impact of germline variants detected in the sequence data.

## Materials and methods

### Patients

All patients diagnosed with PV, ET or PMF according to the 2008 WHO diagnostic criteria (3) in Western Sweden at the Sahlgrenska University Hospital or NU Hospital Group between 2008 and 2013 and reported to the Swedish national blood cancer registry were identified. Basically, all patients in our health care region with suspected MPN are referred to and treated at these two hospitals. Thus, this cohort cover patients in the geographic area without any selection. Of this cohort, 248 patients were included in the study based on informed consent, availability of DNA sample from the time of diagnosis, and review of diagnosis. Data from a subset of this patient cohort have been published previously (12, 13). Details are outlined in **Supplementary Table 1**. Clinical characteristics, clinical course, vitality status, vascular complications, disease transformation and co-existing cancers were collected from the medical records of all patients. Each of the patient’s hospital records were searched after emergency care consultation and hospital admission records that is related to bleeding or thrombotic complications. Follow-up was done from diagnosis until June 2021. The study was performed in accordance with the Declaration of Helsinki after ethical approval.

### Screening for myeloid mutations

Genomic DNA from whole blood from the same sample that was analyzed at diagnosis for the presence of *JAK2*, *CALR* or *MPL* mutation was screened for gene variants in 54 genes or mutational hot spots associated with myeloid malignancies. The TruSight Myeloid Sequencing panel (Illumina FC-130-1010) which also was used for diagnostics in the clinical laboratory at the time of the study, was used, and sequencing was performed on a MiSeq instrument (Illumina) according to manufacturer’s instructions. Secondary analysis was performed with MiSeq Reporter, v.2.4.60.8, using Burrows-Wheeler Aligner mapper and somatic variant caller (Illumina). Data were filtered and mapped to the human genome reference hg19 using Variant studio v3.0 (Illumina), where global filtering was set to >3% and coverage had a minimum of 500 reads. Variants causing missense, frameshift, an altered stop/initiation codon, in-frame insertion/deletion or variants affecting splice site were regarded as mutations. Variants with quality >Q30 and allele frequencies of at least 5% were considered positive for mutation. Known sequencing artefacts and variants previously found in normal controls were excluded from further analysis according to filter strategies used in the clinical laboratory. BAM files from secondary analysis were used to analyze selected variants by Integrative Genomics Viewer (www.broadinstitute.org). Variants in areas with difficult reads were excluded. Previously analyzed data was reanalyzed according to updated bioinformatic settings to make the results comparable regardless of time for sequencing.

### Confirmation of germline variants

Blood sample was taken from patients with variants in *CDKN2A* (rs3731249), *ETV6* (rs145477191), *NOTCH1* (rs61751489) or *MPL* (rs41269541), with a variant allele frequency close to 50%. Blood was enriched for CD3+ cells, using MACS^®^ cell separation kit StraightFrom™ Whole Blood CD3 MicroBeads (Miltenyi Biotech) and Whole Blood Column Kit (Miltenyi Biotech), according to the manufacturer’s protocol. Genomic DNA from CD3+ enriched cells were extracted using QIAamp DNA Blood Mini Kit (Qiagen) and 10 ng of DNA were genotyped using TaqMan SNP genotyping assay (Applied biosciences, Life technologies) according to manufacturer’s protocol. The following assays were used: *CDKN2A* (assay ID: C_25611114_10), *ETV6* (assay ID: C_162058060_10), *NOTCH1* (assay ID: C_90123839_10) and *MPL* (assay ID: ANFV4EK). All samples were analyzed in triplicates using the QuantStudio 3 Real-Time PCR system (ThermoFisher Scientific). Genotypes were determined automatically based on dye component fluorescent emission data depicted in the X–Y scatter plot using Taqman genotyper software v.1.6.0. The gnomAD database v2.1.1 (https://gnomad.broadinstitute.org/) was used to compare frequencies in the MPN cohort with a normal Swedish population.

### Statistical analysis

Fisher’s Exact Test was used to compare differences in frequencies between groups. To estimate overall survival (OS), defined as time from diagnosis to last follow up or death from any cause, the Kaplan Meier Log-rank test was used initially. For multivariable analysis, logistic regression and Cox Regression was used. P-values <0.05 were considered statistically significant. The statistical software used were Analyze-it v.6.15.4 (Microsoft Excel), GraphPad Prism v.9.4.0 and SPSS v29.0.0.0.

## Results

### A population-based cohort

**Figure 1:**
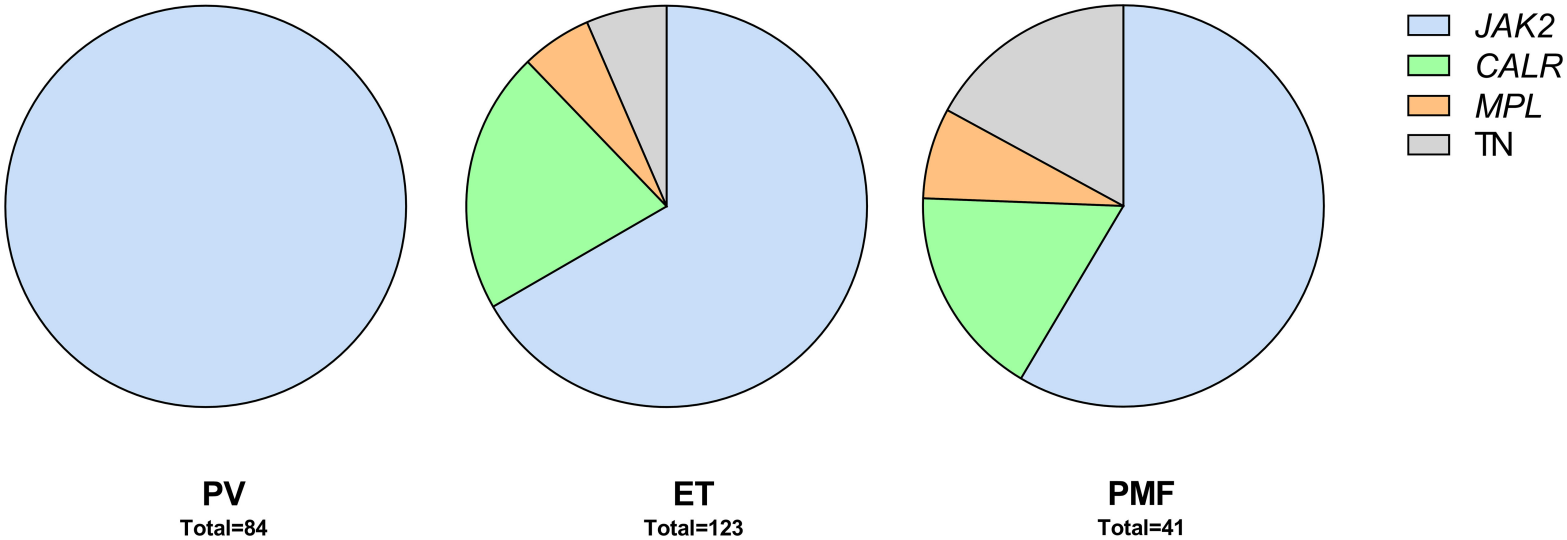
Patients by diagnosis and driver mutations. TN, triple negative.

Between 2008 and 2013, 300 patients were diagnosed with MPN at Sahlgrenska University Hospital and NU Hospital Group in Western Sweden. Of these, 83% (n=248; PV n=84, ET n=123, PMF n=41) were included in the study. All included patients fulfilled the 2008 WHO diagnostic criteria. Age, gender, and blood counts from the time of diagnosis, for the whole MPN group and for the sub entities, are presented in **Table 1**. The distribution of driver mutations found at diagnosis was consistent with expected findings in the different subgroups of MPN (**Figure 1**). One patient with PMF was found to harbor both *JAK2* V617F as well as mutation in the *CALR* gene. Patients not included in the study either declined to participate, had another diagnosis when their medical records were reviewed, or diagnostic material was missing. The median age of these patients was slightly lower (66 years vs. 69 years) but there was no other significant difference between these and the included patients.

**Table 1:** Age, gender and laboratory findings at diagnosis in 248 patients with MPN.

Diagnosis	Age (years)	Gender	Hemoglobin (g/L)	Hematocrit (%)	WBC (10^9^/L)	Platelets (10^9^/L)	EPO (IU/L)
MPN n=248	69 (27-94)	131 F 117 M	145 (60-217)	46 (21-67)	9.5 (1.0-42.7)	689 (5-2412)	4.75 (0.5-40.8)
PV n= 84	70 (37-94)	41 F 43 M	169 (120-217)	53 (37-67)	11.6 (5.2-31.4)	615 (200-2412)	2.1 (0.5-13.2)
ET n=123	68 (27-90)	69 F 54 M	139 (99-170)	43 (31-52)	8.9 (2.9-19.8)	844 (467-2061)	6.1 (1.7-40.8)
PMF n=41	70 (41-85)	21 F 20 M	112 (60-156)	37 (21-49)	8.4 (1.0-42.7)	386 (5-1412)	18 (3.3-578)

Median values with ranges within parenthesis.

### Mutations and survival

**Figure 2:**
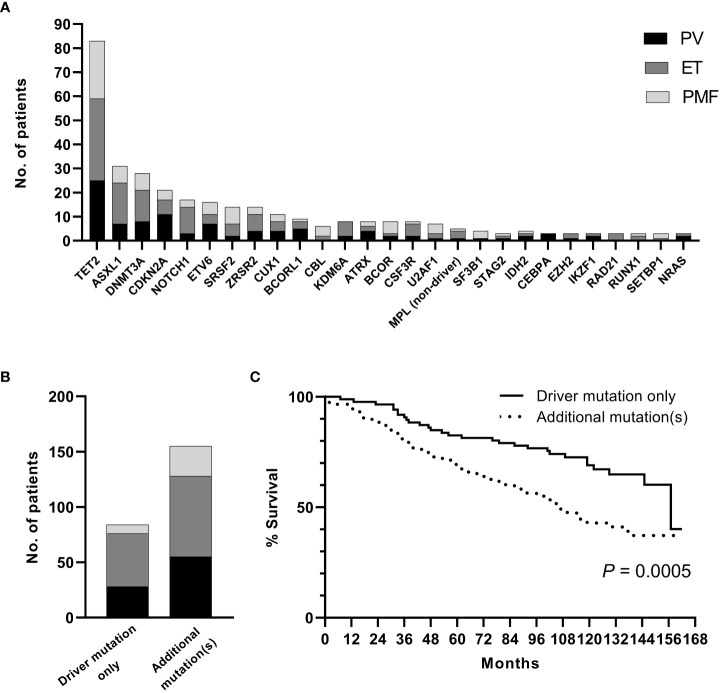
**(A)**. Frequency of additional mutations. **(B)**. Distribution of patients with driver mutation only or addition mutation(s) when confirmed germline variants have been excluded. **(C)**. OS in patients with driver mutation only or with addition mutation(s) (confirmed germline variants excluded).

**Figure 3:**
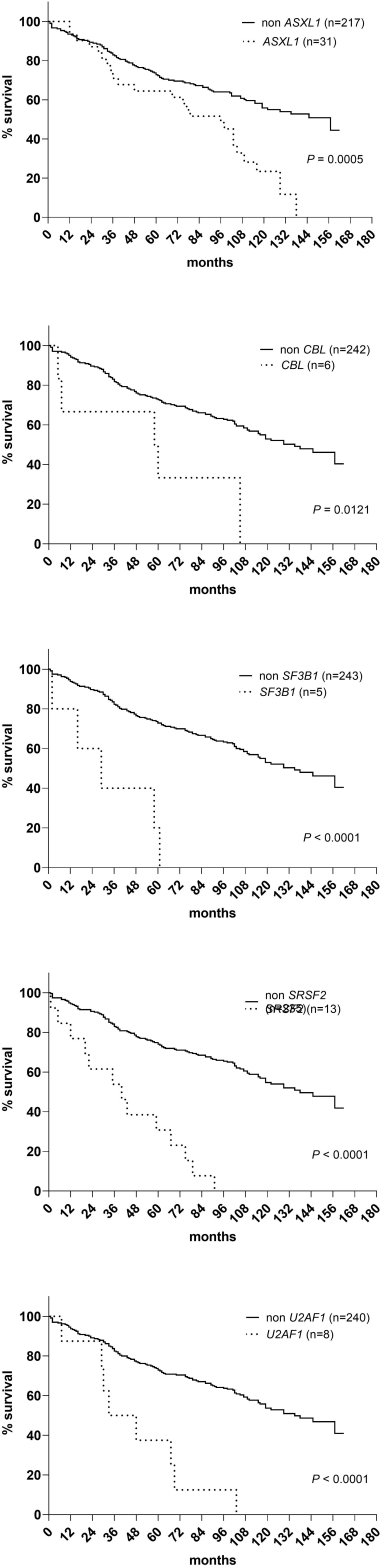
OS in patients with mutations in *ASXL1*, *CBL*, *SF3B1*, *SRSF2* and *U2AF1* respectively according to Kaplan Meier Log-rank test.

A sequencing panel including 54 genes associated with myeloid malignancies was used to screen for genetic variants that could be used as prognostic markers. Variants regarded as mutations other than the diagnostic driver mutations (*JAK2*, *CALR* or *MPL)* were found in 37 genes in at least one patient and in 27 genes in at least three patients (**Figure 2A**; **Supplementary Table 1**). During analysis of the gene data, several recurrent gene variants with allele frequency close to 50% were noted, which implied a hereditary variant. Therefore, analysis of the most common variants *CDKN2A* (NM_001195132.1:c.442G>A), *NOTCH1* (NM_017617.3:c.6853G>A) and *ETV6* (NM_001987.4:c.602T>C) as well as a variant close to a splice site in the *MPL* gene (NM_005373.2:c.1565 + 5C>T) were also analyzed in separated T-cells from new blood samples. This confirmed the variants to be germline. Therefore, these variants were excluded from analyses of prognostic impact. Sixty-three percent of the MPN cases had other mutations in addition to the diagnostic driver mutation (**Figure 2B**). Presence of at least one additional mutation was found to be associated with inferior survival (**Figure 2C**). To investigate if it was mutations in general or mutations in particular genes that had impact on survival, all genes with mutations detected in at least three patients were correlated to survival with the Kaplan Meier Log-rank test. For the whole MPN group, only mutations in five genes correlated significantly with inferior overall survival, *ASXL1* (P=0.0005), *SRSF2* (P<0.0001), *U2AF1* (P<0.0001), *CBL* (P=0.01) and *SF3B1* (P<0.0001) (**Figure 3**). These were further tested with multivariable analysis using Cox regression where also age and type of diagnosis were taken into consideration. When the five genes were grouped together, they still correlated to OS (P=0.002) with a hazard ratio 3.248, and it was not dependent on type of diagnosis (interaction 0.592). Also, age at diagnosis correlated to OS (P<0.001) as expected. However, it should be noted that all cases with *CBL* mutation also harbored mutation in another of the four genes (**Supplementary Table 1**). Therefore, the genes were also tested separately. When these were adjusted for both age and type of diagnosis, only mutations in *SRSF2* and *U2AF1* correlated significantly to OS (**Table 2**).

**Table 2:** Mutation impact on OS in univariate or adjusted (including age and type of diagnosis) analysis.

	Univariate		Adjusted	
	Hazard ratio	P	Hazard ratio	P
*ASXL1*	2.183	<0.001	0.969	0.911
*CBL*	2.987	0.017	1.504	0.533
*SF3B1*	5.459	<0.001	2.525	0.223
*SRSF2*	5.110	<0.001	3.271	<0.001
*U2AF1*	4.491	<0.001	4.991	<0.001

### Mutations and vascular events

Vascular events in MPN are potentially life-threatening. The vascular complications are either thrombosis or bleeding where the co-existence of MPN is a contributing factor. The most common incidences are those that are discovered at the time of diagnosis and the most common thrombotic events were myocardial infarction (n=27), cerebrovascular infarction (n=24), pulmonary embolism (n=19), transient ischemic attack (n=10) and deep vein thrombosis (n=8). The most frequent hemorrhagic complications were gastro-intestinal (n=11) and cerebral bleedings (n=6). Fisher’s Exact Test and logistic regression were used to analyze if *SRSF2* or *U2AF1* which correlated with shorter OS also correlated with occurrence of vascular events before or after diagnosis or in total. However, no such correlation was seen, neither when only the mutated genes were tested, nor when they were combined with age and type of diagnosis.

### Mutations and disease transformation

All MPNs have a risk of transformation into secondary acute myeloid leukemia (AML). In our cohort, 17 cases had transformed to AML. Both mutated genes with correlation to OS were tested with logistic regression. This showed that mutations in *SRSF2* correlated with AML transformation (P=0.002), but this was not the case for *U2AF1* (P=0.236). The analysis was extended to find genes correlated to fibrotic transformation and co-existence with other myeloid hematological malignancies. There were 18 patients that had secondary myelofibrosis transformation from PV and ET. Other myeloid hematological malignancies that co-existed with MPNs included two chronic myelomonocytic leukemia and one myelodysplastic syndrome. Logistic regression showed that mutations in both *SRSF2* and *U2AF1* correlated with co-existing myeloid hematological malignancies (*SRSF2* P=0.05 and *U2AF1* P=0.014).

### Gene germline variants

Identified germ line variants indicated a possible hereditary predisposition of MPN. Comparison of the frequency in our MPN cohort to a normal Swedish population cohort in the gnomAD variant database was performed. Only the variant found in *ETV6* was more frequent in the MPN group (0.0282 vs. 0.00975 in allele frequency). This difference was statistically significant (Fischer’s exact test, p=0.0006). However, there was no correlation between any of the variants and occurrence of early onset MPN. We further used logistic regression to test if any of the variants correlated with occurrence of other cancers (both solid tumors and hematological malignancies outside the MPN group). These cancers occurred about the time and after diagnosis of MPN and were noted upon reviewing the patient´s hospital records. In total, 19 patients with non-hematologic cancers were found. The most common types were colon cancer (n=6) and pancreatic cancer (n=4). However, no significant correlation was seen.

## Discussion

The serious risks all MPNs impose, although at various frequencies, are vascular complications, transformation to more severe hematologic malignancies and ultimately negative impact on OS. It is thus a priority to identify high risk patients in clinical practice. Age at diagnosis as well as occurrence of vascular complications have been reported as risk factors (14, 15). Access to an abundance of genetic data allows genetic profiling to further broaden prognostic information. Mutational status has progressively taken a big role in clinical practice. Occurrence of mutations have also been used to create scoring systems for MPN (16, 17). The initial focus on gene mutations in MPN was on the driver mutations’ importance on disease development. These mutations are found in the genes *JAK2*, *CALR* and *MPL* which all are involved in JAK-STAT signaling (18). Notably, the same *JAK2* mutation is found in both PV, ET and PMF, and mutations in *CALR* and *MPL* are seen in both ET and PMF. Thus, the mutation itself does not seem to determine the MPN phenotype, instead, allele burden has been reported as one factor behind the phenotypic differences (19). The order of acquisition of the driver mutation in relation to additional mutations may also have influence (20, 21). If the *JAK2* mutation precedes mutation in *DNMT3A* or *TET2*, the phenotypic picture would likely be PV. If mutations instead occur in reverse order, the MPN phenotype would likely be ET (22). Host factors also contribute to the development of disease (22, 23). Several predisposing gene variants have been identified that may influence not only the risk of developing disease but also the course of the disease (17, 24–26). It is therefore reasonable to investigate the whole MPN cohort as a group independent of diagnosis when analyzing it from a genetic point of view.

Aside from the driver mutations, several additional mutations have also been reported in MPN. These are subclassified to gene families: epigenetic regulators (*ASXL1, EZH2, TET2, IDH1/2, DNMT3A*), spliceosome (*SRSF2, SF3B1, U2AF1, ZRSR2*), transcriptional regulators (*TP53, RUNX1, IKZF1*), general cell signaling genes (*KRAS, PTPN11*) as well as specific negative regulators of JAK/STAT signaling (*CBL*) (27). Occurrence of additional mutations correlated significantly with inferior OS (**Figure 2C**). Genetic profiling has raised the question if the number of mutations in a particular case is more important for the occurrence of complications or OS rather than in what genes or type of gene the mutations are present. Our results suggest it is not the number of mutations but rather the presence of certain gene mutations that are more informative for prognostic guidance. Mutations in *SRSF2* and *U2AF1* correlated significantly with worse OS in our patient cohort. Although they were more frequent in PMF, which is well known to have an impaired survival compared to patients with PV and ET, mutations in these two genes correlated to worse OS regardless of MPN subtype.

Previous studies, including a subpopulation of our analyzed MPN cohort, have shown that triple negative MPN without *JAK2*, *CALR* or *MPL* mutation have worse prognosis (12, 28). It has also been shown that the presence of several other mutations in addition to a driver mutation correlate with survival (2, 5, 24, 26, 28–32). In this study we initially identified mutations in five non-driver genes (*ASXL1*, *SRSF2*, *U2AF1*, *SF3B1* and *CBL*) to be significantly correlated to impaired OS. Mutations in both *ASXL1* and *SRSF2* have previously been classified as high-risk mutations in both PMF and PV (26, 33). Moreover, mutations in *U2AF1* and *SF3B1* have been identified as genetic risk factors in ET (17). When age at diagnosis as well as type of diagnosis was taken into consideration only mutations in *SRSF2* and *U2AF1* remained associated with shorter OS. Mutations in *CBL* were only found in those patients who harbored mutations in one or more of the four other genes, suggesting that mutated *CBL* might just be a passenger rather than a disease driver. Mutations in *ASXL1* is commonly seen in clonal hematopoiesis, which increases with age (34). This could be an explanation why the presence of *ASXL1* mutation no longer significantly correlated with OS when age was taken into consideration.

Since vascular complications are associated with impaired survival we wanted to investigate if the detected mutations correlated also to vascular events in our MPN cohort. However, neither mutations in *SRSF2* nor *U2AF1* correlated to vascular events before or after diagnosis or in total. Another complication with MPN is transformation to myelofibrosis for PV and ET or to secondary AML for all three MPN. In the present cohort, a significant correlation between mutations in *SRSF2* and transformation to AML was found. Furthermore, mutations in both *SRSF2* and *U2AF1* correlated with transformation from PV and ET to myelofibrosis and development of other hematological malignancies. This is in line with previous findings were mutations in *SRSF2* and *U2AF1* have been reported to serve as prognostic markers for rapid blastic progression in newly diagnosed MPN (35). Moreover, mutations in *SRSF2*, *U2AF1* and *SF3B1* detected at presentation of disease have been associated with rapid fibrotic progression in PMF. This was not demonstrated for mutations in *ASXL1*, *DNMT3A* or *TET2* (36).

Aside from the acquired mutations in our MPN cohort, several specific variants were identified which turned out to be germline. A five- to sevenfold higher risk of MPN among first-degree relatives to MPN patients have previously been reported in Sweden, which suggest a genetic predisposition (37). Also in other myeloid malignancies, the question for germline variants involved in disease have come into focus (38–40). Four variants in our study were more closely investigated, their allele frequency was close to 50%, which could imply a hereditary variant. These genes were *CDKN2A* (NM_001195132.1:c.442G>A), *NOTCH1* (NM_017617.3:c.6853G>A) and *ETV6* (NM_001987.4:c.602T>C) as well as a variant close to a splice site in the *MPL* gene (NM_005373.2:c.1565 + 5C>T). The most frequent occurring *CDKN2A* mutation leading to a p.A148T substitution has been reported as an inherited coding variant associated with leukemic transformation of hematopoietic progenitor cells (41). Comparison of the frequency to a normal Swedish population cohort, however, only revealed the *ETV6* variant to be more common in the MPN patient cohort. This variant did not correlate to earlier onset of disease, which could be expected for an inherited predisposition. On the other hand, in the Landgren study the mean age at diagnosis did not differ between affected relatives and controls (37). The ethical approval of the current study did not include testing of relatives, but it would of course be of interest to see if any of these variants are associated with an increased incidence of hematological or non-hematological malignancies within these families. Nevertheless, it is important to correctly identify germline gene variants to avoid drawing conclusions from non-informative genetic variants but also to provide genetic counseling when called for. In this study we used CD3+ selection of T-cells from collected blood samples to get constitutive DNA, which turned out to be easiest for both referring doctors and gave acceptable DNA yield for the laboratory but might of course misdiagnose somatic variants that are also present in lymphoid cells. Another alternative is a skin biopsy but this may be considered too much of an intervention for some patients.

In conclusion, our study on a population based MPN cohort strengthens previous reports about prognostic value of genetic data in MPN. Thus, a wider gene profiling at diagnosis is of value. In addition, several genetic variants were also identified as germline in this study but gave no obvious clinical relevance. To avoid conclusions from non-informative genetic variants, simultaneous analysis of normal cell DNA from patients at diagnosis should be considered.

## Data availability statement

The original contributions presented in the study are included in the supplementary material, further inquiries can be directed to the corresponding author.

## Ethics statement

The studies involving humans were approved by Etikprövningsmyndigheten (previously research ethics committee at University of Gothenburg, Sweden - Dnr 425-14 and 2021-05747-01). The studies were conducted in accordance with the local legislation and institutional requirements. The participants provided their written informed consent to participate in this study.

## Author contributions

JAs, BA, PJ and LP designed the research study. JAd and JAs performed laboratory work and data analysis. BA, PJ and HP provided patient samples. HP, JAd, JAs, BA, PJ, SN and LP analyzed the combined data and wrote the paper. All authors approved of the final version.
